# Case report: An unusual case of phrenic nerve stimulation in a patient with single chamber implantable cardioverter defibrillator

**DOI:** 10.3389/fcvm.2023.1088697

**Published:** 2023-02-23

**Authors:** Carlo De Innocentiis, Pasquale Astore, Angela Buonpane, Antonia Pia Santamaria, Francesco Patragnoni, Matteo Santamaria

**Affiliations:** ^1^Arrhythmology and Electrophysiology Unit, Gemelli Molise Hospital, Campobasso, CB, Italy; ^2^Agostino Gemelli IRCCS University Hospital Foundation, Rome, Italy; ^3^Cardiac Rehabilitation Unit, Gemelli Molise Hospital, Campobasso, CB, Italy; ^4^Medtronic Italia, Milan, Italy

**Keywords:** phrenic nerve stimulation, cardiac implantable electronic devices, implantable cardioverter defibrillator, cardiac resynchronization therapy, case report

## Abstract

**Background:**

Phrenic nerve stimulation is a well-recognized complication related to cardiac implantable electronic devices, in particular with left ventricular coronary sinus pacing leads for cardiac resynchronization therapy.

**Case presentation:**

We report an unusual case of symptomatic phrenic nerve stimulation due to inadvertent placement of a right ventricular defibrillation lead in coronary sinus posterior branch in a patient with heart failure with reduced ejection fraction with a recently implanted single-chamber cardioverter defibrillator.

**Discussion:**

Phrenic nerve stimulation is a relatively common complication of left ventricular pacing. Inadvertent placement of a right ventricular lead in a coronary sinus branch is a rare but possible cause of phrenic nerve stimulation. Careful evaluation of intraprocedural fluoroscopic and electrocardiographic appearance of pacing and defibrillation leads during implantation may prevent inadvertent placement of a right ventricular lead in the coronary sinus.

## Introduction

Phrenic nerve stimulation (PNS) may complicate up to 30% of cardiac implantable electronic devices (CIEDs) implantation procedures, mainly cardiac resynchronization therapy (CRT) with left ventricular pacing, due to the anatomic contiguity of phrenic nerve to left ventricle lateral wall (1). Although its clinical relevance is limited to a minority of cases, PNS may be responsible for significant symptoms with reduced quality of life and CRT failure (2, 3). Here we report a case of highly symptomatic PNS after single-chamber implantable cardioverter defibrillator (ICD) implantation in a patient with heart failure with reduced ejection fraction (HFrEF).

## Case presentation

**FIGURE 1:**
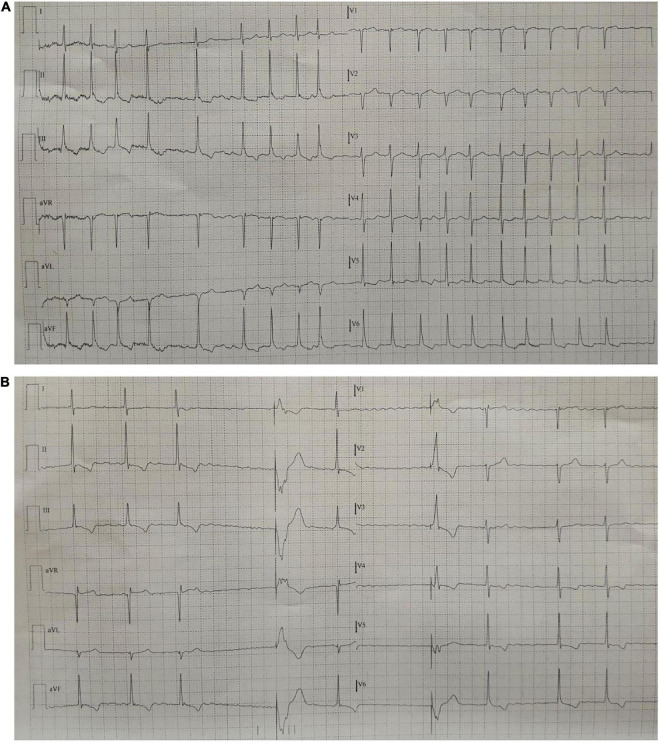
**(A)** 12-lead ECG at admission showing AF with rapid ventricular response despite rate-control therapy. **(B)** 12-lead ECG showing phases of AF with slow ventricular response with ventricular pacing at the lower rate (ICD in VVI 30). Note the morphology of the paced QRS with RBBB pattern, superior axis with R pattern in aVR. AF, atrial fibrillation; ICD, implantable cardioverter defibrillator; RBBB, right bundle branch block.

**FIGURE 2:**
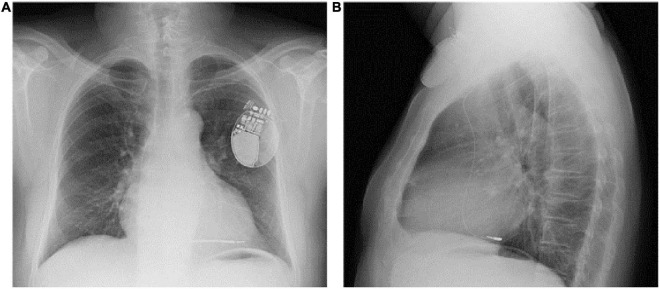
PA **(A)** and LL **(B)** chest x-ray of the patient showing inadvertent lead placement in the posterior branch of the coronary sinus. PA, posteroanterior; LL, laterolateral.

**FIGURE 3:**
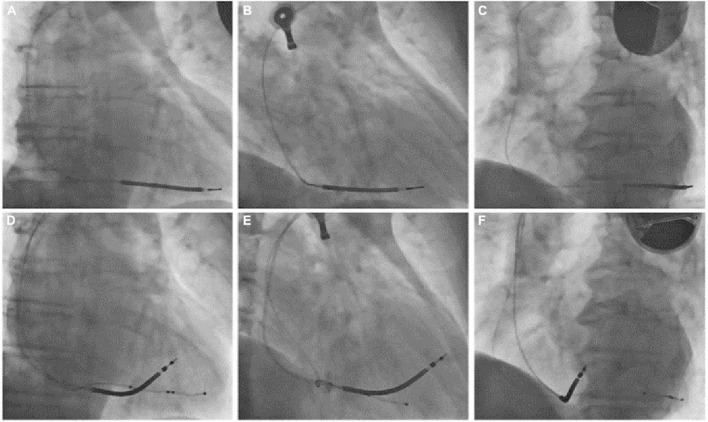
(Superior row) PA **(A)**, RAO 30° **(B)**, and LAO 45° **(C)** fluoroscopic views of the previously implanted single chamber cardioverter defibrillator with a single coil passive fixation defibrillation lead. (Inferior row) PA **(D)**, RAO 30° **(E)**, and LAO 45° **(F)** fluoroscopic views after lead extraction and the reimplantation of a new single-coil active fixation defibrillation lead on the mid portion of the interventricular septum and a coronary sinus quadripolar passive fixation lead for cardiac resynchronization therapy. PA, posteroanterior; RAO, right anterior oblique; LAO, left anterior oblique.

A 68-year-old Caucasian male was referred to our Arrhythmology and Electrophysiology Unit for lead revision of a single-chamber ICD implanted ten months earlier in another center. Two months before ICD implantation, the patient was hospitalized for symptomatic heart failure, NYHA class 3. A 12-lead ECG showed atrial fibrillation (AF) with rapid ventricular response. A transthoracic echocardiography (TTE) demonstrated reduced left ventricular ejection fraction (LVEF, 30%) and severe left atrial enlargement without significant valvular disease. An invasive coronary angiography (ICA) showed multivessel coronary artery disease (MVCAD) with a low Syntax score. Myocardial revascularization with percutaneous coronary intervention (PCI) and two drug-eluting stents (DES) implantation in the left anterior descendent (LAD) coronary artery and the obtuse marginal branch of the left circumflex was performed without complication. The patient was discharged on heart failure and rate-control therapy with a beta-blocker (bisoprolol 5 mg bid), an angiotensin receptor-neprilysin inhibitor (ARNI, sacubitril/valsartan 49/51 mg bid), a mineralocorticoid receptor antagonist (MRA), a loop diuretic, and digoxin (0.125 mg qd). After two months, because of persistent severe left ventricular systolic dysfunction, a single-chamber ICD with a single-coil passive fixation lead was implanted for primary prevention of sudden cardiac death (SCD). During subsequent follow-up, the patient was referred to our center for symptomatic PNS. At admission, the patient presented with mild dyspnea and mild ankle swelling. Blood tests were in the range of normality except for increased levels of N-terminal pro-B-type natriuretic peptide (NT-proBNP, 1,230 pg/ml, nr <125 pg/ml). Device control showed an excessive ventricular pacing percentage (about 15%) although ICD programming in VVI mode with a lower rate of 30 beats per minute, indicating phases of slow ventricular response with the necessity of ventricular pacing. PNS at very low energy output was also confirmed. Several symptomatic episodes of high ventricular rate (HVR) indicated an unsatisfactory pharmacological rate-control despite maximum tolerated drug dosage. Lead parameters were in the range of normality with a normal trend, thus excluding lead failure due to fracture or insulation defect. A 12-lead ECG showed AF with rapid ventricular response and normal QRS duration (90 ms) (Figure 1A). Unfortunately, a 12-lead ECG during ventricular pacing was not recorded before admission, however it was obtained during hospital stay from continuous ECG monitoring (Figure 1B). Paced QRS morphology showed a right bundle branch block (RBBB) pattern and superior axis. A chest X-ray (CXR) was obtained at admission (Figure 2). CXR showed a single-coil passive fixation defibrillation lead placed in coronary sinus posterior branch, thus explaining occasional PNS. Interestingly, the patient previously underwent two other CXR examinations but the incorrect lead placement was not recognized. A TTE was repeated and reduced LVEF without visualization of defibrillation lead in the right ventricle, in the absence of pericardial effusion, was confirmed. Lead explantation and CRT implantation for both high ventricular pacing percentage and predictable subsequent atrioventricular junction (AVJ) ablation for “ablate-and-pace” therapy were planned, because of reported suboptimal rate-control in patient history (alternance of slow and rapid ventricular response). A venogram was performed in order to confirm subclavian vein patency. A successful lead explantation with simple gentle traction using a non-locking stylet was performed. At the same time, we performed implantation of a CRT device with a single-coil active fixation defibrillation lead placed in the mid-ventricular septum, and a quadripolar passive fixation lead in coronary sinus posterior branch (the only available, Figure 3). The LV quadripolar lead was tested for PNS that was absent in pacing configurations excluding distal electrode, with optimal pacing threshold. Bedside echocardiographic examination ruled-out pericardial effusion. The patient was discharged the day after the procedure, and AVJ ablation was postponed after rate control evaluation at subsequent follow-up (Table 1).

**TABLE 1:** Timeline.

Date	Event
12 months prior to admission	HFrEF.
	MVCAD treated with PCI.
	AF with rapid ventricular response.
10 months prior to admission	Single chamber ICD implantation for HFrEF and rate-control therapy.
	Symptomatic PNS during follow-up.
Day 1 admission	Hospital admission for lead revision for symptomatic PNS.
	Radiographic and electrocardiographic evidence of inadvertent lead placement in a coronary sinus branch.
	AF with both slow and rapid ventricular response.
Day 2 admission	Lead explantation and CRT-D implantation for both AF with slow ventricular response and subsequent “ablate-and-pace” therapy.
Day 3 admission	Hospital discharge with planned AVJ ablation for “ablate-and-pace” therapy.

AF, atrial fibrillation; AVJ, atrioventricular junction; CRT-D, cardiac resynchronization therapy-defibrillator; HFrEF, heart failure with reduced ejection fraction; ICD, implantable cardioverter defibrillator; MVCAD, multivessel coronary artery disease; PNS, phrenic nerve stimulation.

## Discussion

**FIGURE 4:**
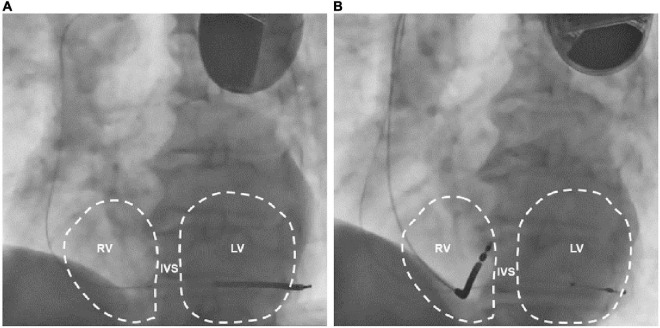
LAO 45° fluoroscopic views of the previously implanted single chamber cardioverter defibrillator **(A)** and the subsequently implanted biventricular cardioverter defibrillator **(B)**. Note that in A the defibrillation lead crosses the midline toward the left ventricle. LAO: left anterior oblique; RV, right ventricle; IVS, interventricular septum; LV, left ventricle.

Phrenic nerve stimulation is a potential CIEDs complication, particularly in case of CRT with left ventricular pacing, due to the anatomic contiguity of the phrenic nerve to the lateral wall of the left ventricle. Direct diaphragmatic stimulation has also been hypothesized for LV pacing leads. PNS was more familiar with unipolar and bipolar coronary sinus leads due to the limited number of pacing configurations with such leads (3, 4). Contemporary quadripolar leads have markedly improved PNS management (5–8). However, in our patient, PNS was related to single-chamber ICD, a less common event. PNS after non-CRT devices implantation was previously reported following RVOT pacing (9) and as a consequence of lead fracture in subclavian crush syndrome (10). We excluded lead failure with fracture or insulation defect because lead parameters were in the range of normality. We excluded ventricular perforation for the same reason and for the absence of pericardial effusion. Therefore, we suspected inadvertent and erroneous lead malposition in a coronary sinus branch, confirmed by CXR. The 12-lead ECG obtained during hospital stay showed a RBBB with superior axis morphology of the paced QRS complexes further corroborating CXR findings (Figure 1B). The exact incidence of inadvertent lead malposition during CIEDs implantation remain unknown and is probably underestimated. A retrospective observational study reported an estimated incidence of inadvertent lead malposition of about 0.3% mainly due to endocardial left ventricular pacing through a patent foramen ovale (PFO), an atrial septal defect (ASD), or inadvertent arterial cannulation (11). A misplaced lead in a coronary sinus branch was the cause of inadvertent lead malposition in only one patient in the study cohort. Our case demonstrates the importance of accurate intraprocedural fluoroscopic and electrocardiographic evaluation of pacing and defibrillation leads during CIEDs implantation, and the necessity of specific training of radiologists in the evaluation of CIEDs related CXR. Anterior oblique fluoroscopic views during CIEDs implantation, particularly left anterior oblique (LAO) view, may prevent the inadvertent placement of a right ventricular lead in the coronary sinus (Figure 4).

## Conclusion

The present case highlights the importance of early recognition of inadvertent placement of a right ventricular lead in a coronary sinus branch, mainly the middle cardiac vein or a posterior branch.

Such complication may be avoided with a careful fluoroscopic and electrocardiographic procedural examination. Both cardiologists and radiologists should be trained to interpret CIEDs’ fluoroscopic appearance. Inadvertent placement of a right ventricular lead in a coronary sinus branch should always be considered in the case of PNS after single or dual chamber pacemaker or ICD implantation.

## Data availability statement

The original contributions presented in this study are included in the article/supplementary material, further inquiries can be directed to the corresponding author.

## Ethics statement

Written informed consent was obtained from all participants for their participation in this study. Written informed consent was obtained from the individual(s) for the publication of any potentially identifiable images or data included in this article.

## Author contributions

CD drafted the manuscript. PA, AB, AS, FP, and MS revised it critically for important intellectual content and given final approval of the version to be published. All authors have been directly involved in patient care, have undertaken patient investigations, and agreed to be accountable for all aspects of the work in ensuring that questions related to the accuracy or integrity of any part of the work are appropriately investigated and resolved.
